# P08-16 Neighborhood walkability and older people's participation in leisure activities

**DOI:** 10.1093/eurpub/ckac095.129

**Published:** 2022-08-29

**Authors:** Essi-Mari Tuomola, Kirsi Keskinen, Taina Rantanen, Erja Portegijs

**Affiliations:** 1 Faculty of Sport and Health Sciences and Gerontology Research Center, University of Jyväskylä, Jyväskylä, Finland; 2 Center for Human Movement Sciences, University Medical Center Groningen, University of Groningen, Groningen, The Netherlands

**Keywords:** Older people, walkability, participation, leisure activities

## Abstract

**Background:**

The neighborhood environment may enhance or restrict older people's opportunities to participate in leisure activities, and thus impact quality of life. Walkability depicts the environment's suitability for walking to different destinations. Little is known concerning about the relation between environment walkability and participation in leisure activities. Our purpose was to study whether neighborhood's objective and perceived walkability were related to participation in various leisure activities outside the home.

**Methods:**

Cross-sectional data of LISPE consisted of 848 community-dwelling people aged 75-90 living in the municipalities of Jyväskylä and Muurame, Finland. Participants' home addresses were geocoded, and walkability index (mixed land-use, street connectivity, and population density) was calculated using geographic information system and categorized into tertiles. From a checklist, participants chose all infrastructure-based facilitators present in their neighborhood, which draw them to outdoor mobility (e.g., good lighting, services close); the sum of these was used as an indicator of perceived walkability. Participation in leisure activities outside the home was self-reported based on frequency and classified as participation (yes/no) in group activities (≥1x/week), physical activity (≥1x/week), and non-group cultural and other activities (≥1x/month). For each leisure activity type, logistic regression models were conducted for walkability index and perceived walkability separately. Analyses were adjusted age, sex, years of education, weekly car use, walking difficulties, and number of chronic conditions.

**Results:**

Logistic regression showed that the older people who lived in areas with high walkability index were more likely to participate in cultural and other activities activity at least once a month (OR = 1.64, 95% Cl = 1.14-2.36) and less likely to participate in physical activity at least once a week (OR = 0.61, 95% Cl = 0.39-0.94, fully adjusted model) than older people living in areas with low walkability index. Older people reporting higher perceived walkability participated more often in physical activity than those who reported lower walkability (OR = 1.14, 95% Cl = 1.05-1.23).

**Conclusions:**

Living in the area of high walkability enhanced participation in cultural activities and decreased participation in physical activity. However, higher perceived walkability may motivate older people to be physically active. Environment which offers good infrastructure for outdoor mobility provides opportunities to participate in specific leisure activities.

